# Autonomy‐Supportive Circuit Training Program: Differential effects on adaptive behavior and emotional symptoms in children with and without autism spectrum disorder

**DOI:** 10.1002/pcn5.70205

**Published:** 2025-09-19

**Authors:** Yukari Murakami, Koki Tanida, Takayuki Munechika, Satoshi Kurose, Yutaka Kimura

**Affiliations:** ^1^ Health Science Center Kansai Medical University Hirakata, Osaka Japan; ^2^ AKIDS Lab Osaka, Osaka Japan; ^3^ Wago Hospital Togo, Aichi Japan; ^4^ Faculty of Sport and Health Sciences Osaka Sangyo University Daito, Osaka Japan

**Keywords:** autism spectrum disorder, behavior, exercise, self‐regulation, Strength and Difficulties Questionnaire

## Abstract

**Aim:**

This prospective non‐randomized controlled study examined the Autonomy‐Supportive Circuit Training Program (ASCTP), a structured physical activity intervention. The primary objective was to assess and compare changes in internalizing (emotional symptoms) and externalizing (behavioral difficulties) traits between children with autism spectrum disorder (ASD) and those with subthreshold ASD following ASCTP participation.

**Methods:**

A total of 28 boys (aged 4–10 years) were divided into an ASD (*n* = 14) and a non‐ASD group (*n* = 14). The ASCTP intervention was developed and implemented in an exercise‐focused daycare center for children with disabilities in Japan. The intervention consisted of a structured 45‐min exercise program conducted once a week for 6 months. It emphasized an autonomy‐supportive and standardized session structure, implemented by trained facilitators, and was specifically designed to support children's self‐regulation and individualized activity selection. Emotional and behavioral traits were assessed pre‐ and post‐intervention using the Strengths and Difficulties Questionnaire, completed by the parents. Additionally, the children's lifestyle habits (sleep, screen time, and breakfast consumption) were recorded pre‐ and post‐intervention through parental reports.

**Results:**

The ASD group showed a significant reduction in behavioral difficulties, whereas the non‐ASD group exhibited an increase in behavioral difficulties (−1 [−2 to 0] vs. 1 [−1 to 1], adjusted *p* = 0.034, *r* = 0.45). Although no significant changes in emotional symptoms were present in either group, a decreasing trend was observed.

**Conclusion:**

Participation in the ASCTP was associated with a reduction in behavioral difficulties in children with ASD, which may reflect the potential benefits of the structured framework of the program. Conversely, the increased behavioral difficulties observed in the non‐ASD group may indicate the need for more flexible intervention approaches.

## INTRODUCTION

Difficulties with social communication, repetitive behavior, and restricted interest primarily characterize autism spectrum disorder (ASD). In addition to children diagnosed with ASD, many children with subclinical ASD traits or other neurodevelopmental characteristics may exhibit comorbid internalizing problems, such as anxiety and depression, as well as externalizing problems, such as impulsivity and aggression.^1^, ^2^ Even in children with ASD without intellectual disabilities or ASD traits, these co‐occurring symptoms can affect daily life, exacerbating difficulties in social adaptation and emotional regulation.^3^ If these issues are not appropriately addressed, they may lead to further declines in academic performance and quality of life, increased family stress, and reduced opportunities for social inclusion.

Existing interventions for children with ASD primarily focus on improving core ASD symptoms through behavioral therapy.^4^ Although these approaches have a certain level of scientific support, they often fail to sufficiently address emotional and behavioral problems that significantly affect daily life.^5^ Furthermore, effective intervention methods for children who do not meet the diagnostic criteria for ASD but experience social and emotional difficulties remain underexplored. Given this background, there is an increasing need for interventions that address both internalizing and externalizing symptoms and support both children with ASD and those with neurodevelopmental characteristics but without an ASD diagnosis.

Physical activity has been widely recognized as an effective intervention for improving the social skills and physical health of children with ASD.^6^, ^7^, ^8^, ^9^, ^10^, ^11^ However, conventional programs are often instructor‐led, following predetermined exercises that may not fully accommodate individual interests and motivations. In addition, the effects of physical activity on internalizing and externalizing symptoms in children with different neurodevelopmental characteristics have not been sufficiently examined. While leveraging the restricted interests of children with ASD has been proposed as a strategy for enhancing engagement in the intervention,^12^, ^13^ structured exercise programs that systematically incorporate this approach remain limited.

To address these challenges, this study developed and evaluated an “Autonomy‐Supportive Circuit Training Program (ASCTP).” ASCTP is a structured exercise intervention program that integrates autonomy support while maintaining an organized framework. By engaging in activities that align with their interests, children are encouraged to actively participate in physical activities while promoting autonomy and behavioral flexibility.

This study employed a prospective non‐randomized controlled design to explore the associations between participation in ASCTP and changes in behavioral characteristics of both ASD and non‐ASD children, particularly concerning internalizing and externalizing symptoms. By including a comparison group of children without ASD, this study aimed to examine whether these associations were specific to children with ASD or applicable to a broader range of children with neurodevelopmental characteristics. Ultimately, this study explores ASCTP as a potential new intervention model contributing to children's well‐being with diverse developmental profiles.

## METHODS

### Participants

**Figure 1:**
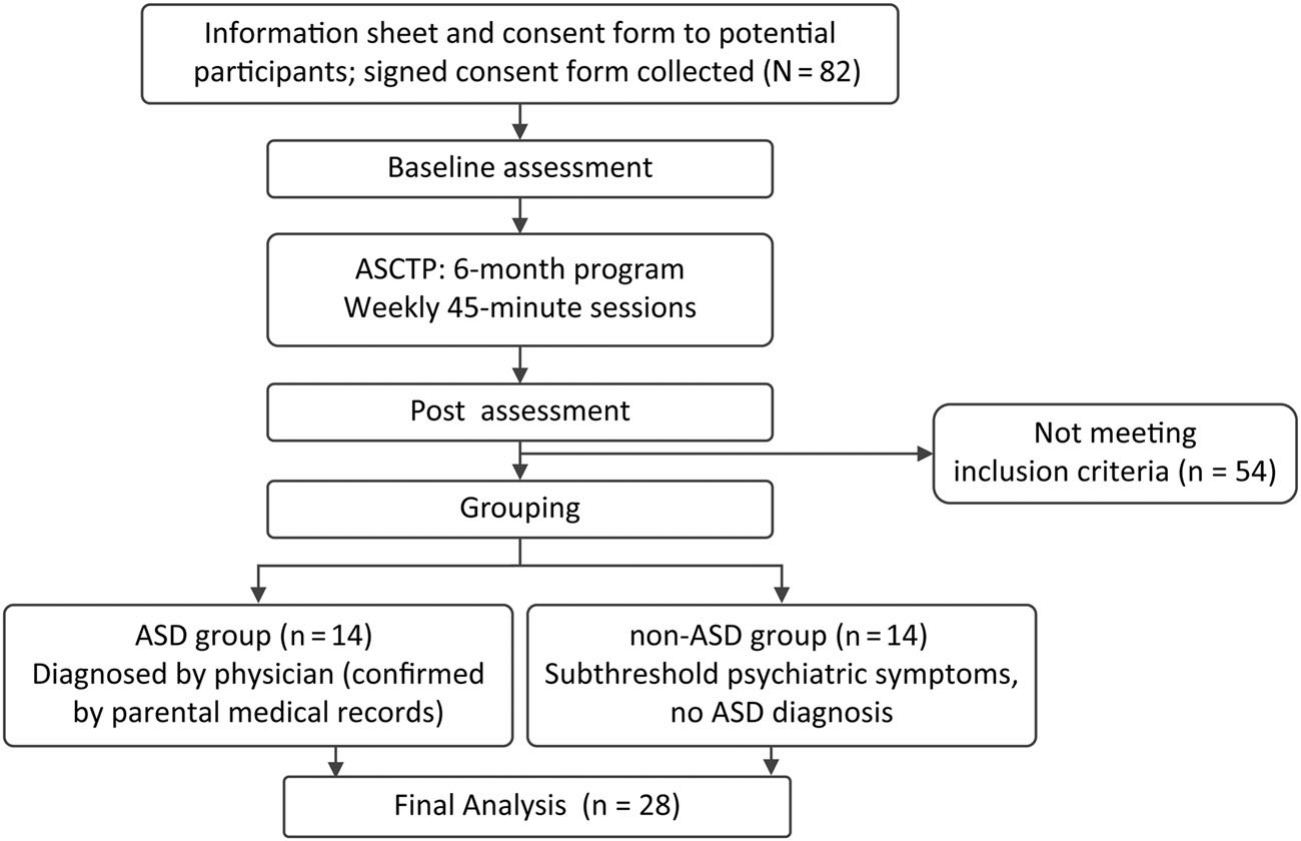
Flowchart of participant enrollment and grouping. Eighty‐two participants completed the baseline assessment. After a 6‐month Autonomy‐Supportive Circuit Training Program (ASCTP), post‐assessment was conducted. Twenty‐eight participants were included in the final analysis (autism spectrum disorder [ASD] group, *n* = 14; non‐ASD group, *n* = 14).

The participants were recruited through the daycare facility staff and informational flyers provided to their families. As presented in Figure 1, among the 82 children who consented to participate, 28 boys (aged 4–10 years, mean age 7.0 ± 1.8 years) were included in the analysis after excluding those with moderate to severe intellectual disabilities, those diagnosed with attention‐deficit hyperactivity disorder (ADHD), and those taking psychotropic medications. Participants were divided into two groups. The ASD group (*n* = 14) comprised children diagnosed with ASD by a physician, as confirmed by medical records. The non‐ASD group (*n* = 14) comprised children who had not been formally diagnosed with ASD but were deemed eligible for welfare‐based developmental support services based on physician statements submitted during the local government application process. These children were considered gray‐zone cases, often presenting with subclinical features such as mild anxiety, attention deficits, impulsivity, or emotional dysregulation. The exact distribution of these features was not systematically recorded. This study was conducted following the Declaration of Helsinki, and ethical approval was obtained from Ichimoto Inc. Ethics Review Board approval (TRAQ#202301).

### Outcome measures

The primary outcome measure was the Strengths and Difficulties Questionnaire (SDQ), a widely used behavioral screening tool developed by Goodman.^14^ The SDQ was selected for its brevity and ability to assess internalizing and externalizing symptoms, making it a practical assessment tool for this study. It is commonly employed in intervention studies to track behavioral changes over time.^15^, ^16^ Unlike other diagnostic tools such as the Autism Diagnostic Observation Schedule (ADOS), which is primarily used for diagnostic purposes rather than detecting intervention effects, the SDQ is well‐suited for longitudinal assessments of behavioral changes.^17^, ^18^

The SDQ comprises 25 items divided into five subscales: emotional symptoms, conduct problems, hyperactivity/inattention, peer relationship problems, and prosocial behavior. Each item is rated on a three‐point scale: “not true” (0), “somewhat true” (1), and “certainly true” (2). The total difficulty score was derived from the first four subscales, with higher scores indicating greater behavioral difficulties. The SDQ has been translated into multiple languages and is widely used in various countries.^19^, ^20^, ^21^, ^22^, ^23^, ^24^ The Japanese version of the SDQ was translated and standardized in 2008.^19^

We used the subscales of emotional symptoms and conduct problems to assess internalizing and externalizing symptoms, respectively. Previous research has demonstrated that these subscales reflect the major symptom categories of pediatric psychiatric disorders.^25^ Furthermore, comparisons with the Child Behavior Checklist (CBCL) have confirmed that these subscales appropriately differentiate between internalizing and externalizing symptoms.^26^, ^27^, ^28^ Guardians completed the Japanese version of the SDQ^19^ before and after the intervention.

Children's lifestyle habits were evaluated as the secondary outcome measures. Regarding sleep habits, guardians answered three questions concerning whether their child (1) wakes up early, (2) maintains a consistent wake‐up time on weekdays and weekends, and (3) avoids staying up late. A score of 1 point was assigned for each “No” response, yielding a total score ranging from 0 to 3, with higher scores indicating irregular sleep habits.

To assess screen time, guardians responded to the question, “How much screen time (TV, video games, smartphones, etc.) does your child have per day?” Responses were classified into three categories: “less than 30 min” (1 point), “less than 2 h” (2 points), and “more than 2 h” (3 points), with higher scores indicating longer screen usage.

The guardians answered the question, “Does your child eat breakfast every day?” with three response options: “always,” “sometimes skips,” and “never eats breakfast.” The proportion of children in the latter two categories was used to indicate the frequency of breakfast consumption. These secondary outcome measures were assessed before and after the intervention, following the same procedure.

### Intervention

The intervention was conducted exclusively within a daycare facility. No additional treatments or interventions were introduced within the research facility; however, some participants may have received other treatments or participated in physical activity programs at other institutions. These external activities were not monitored or controlled in this study.

The intervention program consisted of 45‐min exercise sessions conducted once per week for 6 months. Each session followed a structured format including an opening (greeting and goal setting), warm‐up, main activities, cool‐down, and cleanup. The program emphasizes individualized support, creates an environment that encourages voluntary participation, and enhances motivation for physical activity (for an example of exercise program flow, see Appendix).

Children were allowed to select their activities at the beginning of each session. Available options include balance disks, mini‐hurdles, stepping stones, trampolines, vaulting boxes, mats, horizontal bars, balance balls, various types of balls (e.g., ping‐pong balls, basketballs, and softballs), boulders, and jump ropes. To accommodate children with language comprehension difficulties, activity instructions were provided using visual aids such as picture cards and demonstrations.

The session commenced with salutations and goal confirmation, followed by a warm‐up incorporating animal‐mimicking exercises designed to activate the core and promote coordination. Each main activity lasted approximately 8 min, and upon completion, children received immediate reinforcement in the form of a “flower stamp” (praise stamp) from the instructor, based on the core principles of Applied Behavior Analysis (ABA) to enhance their sense of achievement. The activities were designed without competitive elements to accommodate children who preferred to work at their own pace. The cooldown phase included stretching exercises to promote relaxation, and children were encouraged to participate in tidying up by assigning roles, such as organizing equipment or assisting instructors. This approach fosters intrinsic motivation by allowing children to recognize their contributions rather than relying on external control.

The program was tailored to the interests of each child. For example, for children interested in trains, a course was designed using balance disks as “trains” to enhance motivation. In exercises using balance stones or agility ladders, instructors demonstrate the activity and then guide the children to design their courses, thereby fostering autonomy. Instructor involvement was adjusted according to each child's developmental stage to enable independent exercise participation.

The program also adjusted the exercise intensity and difficulty based on each child's developmental stage to prevent excessive strain. Appropriate hydration was encouraged throughout the activities to prevent fatigue and stress accumulation. While most activities were conducted individually, some, such as ball throwing and jump rope exercises, were performed in pairs or small groups to promote social skill development.

Thus, this exercise program aimed to provide appropriate support tailored to each child's developmental characteristics, while respecting their autonomy. To further support standardization while maintaining individualized elements, all facilitators received structured training before the program began. This training covered the guidance on standardized session flow (opening, warm‐up, main activities, cool‐down, and cleanup), safety management, use of visual supports, strategies for promoting children's autonomous choice, and consistent application of reinforcement techniques such as praise stamps based on ABA principles. A detailed session manual was also provided, outlining the standardized sequence of session components, example activities for each phase, strategies for managing activity choices within predefined options, methods for adjusting intensity according to each child's developmental stage, and including example visual support cards and standardized reinforcement phrases to promote consistent implementation. The sessions were conducted under the clinical supervision of the principal investigator to ensure intervention fidelity.

### Statistical analysis and measurements

The total scores of the SDQ subscales (emotional symptoms and conduct problems) were calculated, and changes in scores before and after the intervention were examined using median and interquartile range (IQR) due to the non‐normal distribution of the data. Additionally, median and IQR were obtained for sleep and screen time scores, and the frequency of breakfast consumption was analyzed as a percentage. Data from all 28 participants were analyzed using JMP Pro 17 (SAS Institute Inc.).

### Baseline group differences

Since normality and homogeneity of variance assumptions were met, Student's *t*‐test was used to compare baseline SDQ scores (emotional symptoms and conduct problems), as well as continuous demographic variables such as age, height, body weight, Rohrer index, and birth weight, between the ASD and non‐ASD groups. For categorical variables including presence of mild intellectual disability, family structure (single‐parent or with siblings), ongoing hospital visits, and preterm birth, Fisher's exact test was applied. All tests were two‐tailed, and a *p*‐value < 0.05 was considered statistically significant.

### Intervention effects (within‐group comparisons)

Within each group, changes in SDQ scores (emotional symptoms and conduct problems), sleep scores, and screen time scores before and after the intervention were analyzed using the Wilcoxon signed‐rank test. Adjusted *p*‐values for the two SDQ subscales (primary outcomes) were calculated with Bonferroni correction. Effect sizes (*r*) for the pre–post changes in SDQ scores were calculated by dividing the *Z*‐value by the square root of *N*. The interpretation of *r* values followed conventional benchmarks: small (0.10 ≤ *r* < 0.30), moderate (0.30 ≤ *r* < 0.50), and large (*r* ≥ 0.50) effects.^29^ Changes in breakfast consumption were assessed using McNemar's test. Results for sleep, screen time, and breakfast variables were treated as exploratory and interpreted at a nominal significance level of *p*  <  0.05, without adjustment for multiple comparisons.

### Between‐group comparisons of score changes

The Mann–Whitney *U* test was used to compare the changes in emotional symptoms, conduct problems, sleep scores, and screen time scores between the ASD and non‐ASD groups. Bonferroni correction was applied for the two SDQ subscales (primary outcomes). Effect sizes (*r*) for the between‐group differences in SDQ change scores were calculated by dividing the *Z*‐value by the square root of *N* and interpreted as small (0.10 ≤ *r* < 0.30), moderate (0.30 ≤ *r* < 0.50), and large (*r* ≥ 0.50) effects.^29^ Fisher's exact test was used to analyze changes in breakfast consumption. Results for sleep, screen time, and breakfast variables were treated as exploratory and interpreted at a nominal significance level of *p*  <  0.05, without adjustment for multiple comparisons.

## RESULTS

The participant characteristics are presented in Table 1. No significant differences were observed between the ASD and non‐ASD groups in terms of age, height, body weight, Rohrer index, presence of mild intellectual disability, family structure, history of continuous medical visits, preterm birth, or birth weight. However, three participants in the ASD group had a Rohrer index of 160 or higher, classifying them as obese and highlighting potential concerns regarding physical health.

**Table 1:** Baseline characteristics of the participants.

Characteristics	ASD (*n* = 14)	non‐ASD (*n* = 14)	*p* value
Age (years)	7.2 (1.6)	6.9 (1.9)	0.600
Height (cm)	122 (11.0)	122 (12.0)	0.868
Body weight (kg)	24.7 (7.7)	22.2 (5.0)	0.316
Rohrer index	133 (22.2)	124 (18.2)	0.239
Mild intellectual disability, *n* (%)	2 (14)	0 (0)	0.482
Family structure (single‐parent/with siblings), *n* (%)	2 (14)/7 (50)	0 (0)/5 (36)	0.482/0.703
Ongoing hospital visits, *n* (%)	10 (71)	4 (29)	0.057
Preterm birth, *n* (%)	1 (7)	4 (29)	0.325
Birth weight (g)	2975.5 (269.3)	2968.1 (213.3)	0.952

*Note*: Values are presented as mean (standard deviation, SD) or number (%). All participants used the daycare facility for 6 months. *p* values were calculated using Student's *t*‐tests for continuous variables and Fisher's exact test for categorical variables. A significance level of *p* < 0.05 was considered.

Abbreviation: ASD, autism spectrum disorder.

Additionally, there were no significant differences between the ASD and non‐ASD groups in emotional symptoms (*t* = −1.93, df = 22, *p* = 0.067) or conduct problems (*t* = −1.43, df = 24, *p* = 0.166) at baseline.

**Figure 2:**
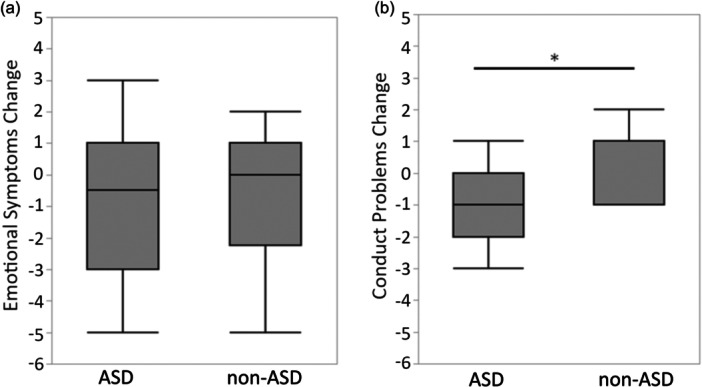
Changes in Strengths and Difficulties Questionnaire (SDQ) scores before and after the intervention. (a) Emotional symptoms. (b) Conduct problems. For box plots, the horizontal line represents the median, the box represents the interquartile range, and the whiskers indicate the data range. Between‐group comparisons of change scores were assessed using the Mann–Whitney *U* test, with Bonferroni correction for multiple comparisons. The reduction in conduct problems was significantly greater in the autism spectrum disorder (ASD) group than in the non‐ASD group (adjusted *p* = 0.034), meeting the threshold of *p* <  0.05; no significant between‐group difference was observed for emotional symptoms. An asterisk (*) indicates a significant group difference at *p* < 0.05.

Regarding the SDQ scores, the ASD group exhibited a significant reduction in conduct problems post‐intervention (adjusted *p* = 0.048, *r* = 0.67), whereas no significant change was observed in the non‐ASD group (adjusted *p* = 1.000, *r* = 0.24). Conversely, emotional symptoms showed no significant changes in either group (ASD: adjusted *p* = 0.768, *r* = 0.35; non‐ASD: adjusted *p* = 1.000, *r* = 0.29) (Table 2). A comparison of score changes between the groups revealed that the ASD group demonstrated a significantly greater reduction in conduct problems than the non‐ASD group (adjusted *p* = 0.034, *r* = 0.45). However, no significant between‐group difference was found for changes in emotional symptoms (adjusted *p* = 1.000, *r* = 0.05) (Figure 2).

**Table 2:** Changes in Strengths and Difficulties Questionnaire (SDQ) subscale scores before and after the intervention (median [interquartile range (IQR)]).

Outcomes	Group	Pre	Post	*p* value (raw)	*p* value (adjusted)	*r*
Emotional symptoms	ASD	6 [5–7]	5 [4–7]	0.192	0.768	0.35
	non‐ASD	4 [3–6]	4 [2–6]	0.311	1.000	0.29
Conduct problems	ASD	4 [3–4]	3 [2–4]	0.012	**0.048**	0.67
	non‐ASD	3 [2–4]	4 [3–4]	0.381	1.000	0.24

*Note*: Statistical significance was assessed using the Wilcoxon signed‐rank test. Adjusted *p*‐values were calculated with Bonferroni correction. Statistically significant results are shown in bold, with a significance level of *p* < 0.05. Effect sizes (*r*) were calculated by dividing the *Z*‐value by the square root of *N*.

Abbreviation: ASD, autism spectrum disorder.

Regarding lifestyle factors, while non‐significant trends were observed in the ASD group toward lower sleep scores and more frequent breakfast consumption, no statistically significant changes were found in any lifestyle measure in either group (Table 3).

**Table 3:** Changes in sleep, screen time, and breakfast consumption before and after the intervention (median [interquartile range (IQR)] or *n* [%]).

Outcomes	Group	Pre	Post	*p* value (pre–post)	*p* value (between groups)
Sleep score	ASD	0 [0–2]	0 [0–1]	0.125	0.214
	non‐ASD	1 [0–2]	1 [0–2]	1.000	
Screen time score	ASD	3 [2–3]	3 [2–3]	0.250	0.954
	non‐ASD	2 [2–3]	2 [1–3]	0.750	
Breakfast consumption, *n* (%)	ASD (always/sometimes or never)	11 (78.6)/3 (21.4)	13 (92.9)/1 (7.1)	0.157	1.000
	non‐ASD (always/sometimes or never)	14 (100)/0 (0)	14 (100)/0 (0)	NA	

*Note*: Sleep scores were based on parental responses to three questions regarding wake‐up time and sleep habits. Higher scores indicate poorer sleep quality. Screen time scores were based on parental responses regarding weekday screen use; higher scores indicate longer screen time. Pre–post comparisons within groups were analyzed using the Wilcoxon signed‐rank test, and between‐group comparisons of score changes were analyzed using the Mann–Whitney *U* test. For all sleep, screen time, and breakfast variables, results were considered exploratory and interpreted at a nominal significance level of *p* < 0.05 without adjustment for multiple comparisons. McNemar test was not applicable to breakfast consumption because all participants in the non‐autism spectrum disorder (non‐ASD) group consistently consumed breakfast.

Abbreviations: ASD, autism spectrum disorder; NA, not applicable.

## DISCUSSION

### Clinical implications

The findings of this study demonstrated a significant reduction in conduct problems in children with ASD, accompanied by a moderate effect size. This aligns with previous research suggesting that physical activity enhances executive function, motor coordination, and social interaction, ultimately improving behavioral regulation.^8^, ^30^ These results support the efficacy of the ASCTP intervention in addressing externalizing symptoms (e.g., impulsivity and aggression) commonly observed in children with ASD. Nevertheless, these results should be interpreted with caution due to the small sample size of the study.

A distinctive feature of the ASCTP intervention is incorporating a self‐selection mechanism that encourages active engagement and increased motivation. Children with ASD often experience stress when faced with unpredictable situations or when engaged in uninteresting activities. However, the structured framework of the ASCTP, which allowed participants to choose movement tasks within predefined time limits and repetition cycles, likely provided a sense of security and facilitated self‐regulation. Furthermore, the ASCTP may have promoted social interaction by incorporating cooperative cleanup tasks, enforcing turn‐taking rules, and fostering joint attention within a structured program.

Additionally, physical activity has been reported to exert a regulatory effect on behavior through neurophysiological mechanisms, such as stress reduction and autonomic nervous system modulation.^31^ In the present study, exercise‐induced moderate physical fatigue may have contributed to suppressing oppositional behavior and impulsivity, thereby reducing externalizing symptoms. These findings suggest that implementing the ASCTP within developmental support facilities and special education programs in schools could be an effective strategy for managing behavioral problems in children with ASD. However, because allocation was not randomized, the findings should be interpreted as associations rather than causal effects. Selection bias and unmeasured confounding—such as baseline motor ability, family environment, and concurrent therapies—may have contributed to the observed reductions in behavioral difficulties. Conversely, in the non‐ASD group, there was a tendency toward increased behavioral difficulties post‐intervention. This may reflect the differences in how neurodevelopmental profiles interact with structured interventions. For children without ASD, the rigid structure and predefined sequences of the ASCTP may conflict with their preference for flexibility, inadvertently inducing stress. Alternatively, the observed increase in behavioral challenges might be attributed to heightened parental awareness. Given that parental evaluations were used to assess behavioral changes in this study, increased attention to conduct problems post‐intervention may have led to a higher frequency of reported behavioral issues, introducing a potential measurement bias that warrants further investigation.

Moreover, the heterogeneity within the non‐ASD group (comprising children with various subclinical characteristics, such as subclinical anxiety, inattention, and impulsivity) may have contributed to the diverse responses to the intervention. This variability may have led to individual differences in response to the intervention and in changes in behavioral rating scores, thereby complicating the interpretation of the results. While the ASCTP incorporated elements of flexibility (e.g., activity selection and creative movement tasks), these features may not have fully accommodated the diverse motivational profiles within this group. Some children may have preferred activities emphasizing teamwork, whereas others may have benefited more from competitive elements. Thus, future interventions should assess cognitive and behavioral traits before implementation and tailor the programs accordingly. Expanding the range of activity choices to align with individual motivational factors may enhance the effectiveness of physical activity programs.

### Emotional symptoms

Both the ASD and non‐ASD groups tended to reduce emotional symptoms post‐intervention; however, these changes did not reach statistical significance. From the perspective of Self‐Determination Theory (SDT),^32^ it is possible that this trend reflects the program's emphasis on fostering the theory's three fundamental psychological needs: *autonomy*, *competence*, and *relatedness*.

*Autonomy* was supported by allowing children to select from a variety of activities at the beginning of each session, as well as by incorporating opportunities for creative expression—such as designing their own obstacle courses or choosing movement sequences—which enhanced their sense of volitional engagement. *Competence* was fostered through appropriately scaled physical challenges and the use of immediate, positive reinforcement (e.g., praise stamps) to celebrate individual achievements, thereby promoting mastery and self‐efficacy. *Relatedness* was cultivated through structured social interactions, such as paired exercises and supportive instructor‐child communication, which helped children feel understood, respected, and emotionally connected within the group context. According to SDT, satisfaction of these three needs facilitates intrinsic motivation and emotional stability.^32^

However, the lack of statistically significant changes highlights the limitations of relying solely on physical activity to address emotional challenges. This underscores the need for an interdisciplinary approach that integrates structured physical activity with psychological support to address emotional regulation comprehensively. Incorporating cognitive behavioral therapy (CBT) or social‐emotional learning (SEL) interventions that specifically target emotion regulation strategies may complement physical activity programs. Additionally, mindfulness‐based techniques, such as guided relaxation exercises, mindful movement activities, and brief mindfulness breaks, may enhance emotional outcomes.

Collaboration between healthcare, welfare, and educational professionals to develop integrated programs that combine exercise with psychological support could provide more effective interventions. Furthermore, previous studies have reported that parental involvement is associated with reductions in anxiety in children with ASD.^33^ Providing training for parents and educators to implement autonomy‐supportive physical activities in the home and school settings may further enhance intervention efficacy.

### Lifestyle factors

Finally, the lack of statistically significant changes in sleep quality, breakfast consumption, and screen time highlights the necessity of a multifaceted, long‐term support strategy incorporating family environment and parental involvement to facilitate sustainable behavioral modifications.

### Limitations

This study has some limitations. First, because the SDQ is a subjective assessment questionnaire, parental evaluation bias may have influenced the results. Second, the non‐randomized design introduces the risk of selection bias and unmeasured confounders, such as age, ASD severity, baseline motor ability, family dynamics, school environment, access to other therapies, and cognitive capacity, which may have influenced the observed outcomes and limit causal interpretations. Third, the small sample size limited the generalizability of the findings. Finally, the sustainability of behavioral changes remains uncertain due to the absence of long‐term follow‐up assessments.

## CONCLUSIONS

ASCTP participation was associated with reductions in behavioral problems in children with ASD, suggesting a link to the regulation of externalizing symptoms. In contrast, an increasing trend in behavioral difficulties was observed in the non‐ASD group, which may reflect differences in interactions with structured interventions. Although emotional symptoms showed a decreasing trend in both groups, no statistically significant changes were detected, highlighting the need for an approach that integrates psychological support. Similarly, the lack of significant changes in lifestyle habits underscores that long‐term support is required for sustained behavioral changes. These findings support the potential benefits of exercise‐based interventions for children with ASD while emphasizing the importance of individualized support.

## AUTHOR CONTRIBUTIONS

**Yukari Murakami**: Conceptualization; methodology; data analyses; original writing; editing. **Koki Tanida**: Participant recruitment; data collection; editing. **Takayuki Munechika**: Conceptualization; methodology; editing. **Satoshi Kurose**: Data analysis and editing. **Yutaka Kimura**: Methodology and editing. All the authors have read and approved the final version of the manuscript.

## CONFLICT OF INTEREST STATEMENT

The authors declare no conflicts of interest.

## ETHICS APPROVAL STATEMENT

This study was conducted in accordance with the Declaration of Helsinki and was approved by Ichimoto Inc. Ethics Review Board (TRAQ#202301).

## PATIENT CONSENT STATEMENT

Written informed consent was obtained from the parents of all participants.

## CLINICAL TRIAL REGISTRATION

This study was conducted as an in‐house support activity within a facility and does not require clinical trial registration. The intervention was a small‐scale support activity that did not meet the criteria for a formal clinical trial.

## Data Availability

The data that support the findings of this study are available on request from the corresponding author. The data are not publicly available due to privacy or ethical restrictions.

## Appendix

See Table A1.

**Table A1:** Example of the exercise program flow.

Step	Example 1	Example 2	Example 3
Opening	Children greet each other and express readiness	Same as Example 1	Same as Example 1
Goal setting and activity selection	Set a goal and choose three activities from at least 10 options	Same as Example 1	Same as Example 1
Warm‐up	Animal imitation exercises	Same as Example 1	Same as Example 1
Activity 1	Balance course creation and walking	Ninja training on the mat (forward roll, backward roll, handstand like a ninja)	Cone maze run (weave through the cones like a race car driver)
Activity 2	Bouldering challenge (climbing to collect dinosaur stickers at various heights)	Racket rally (hit a ball or a balloon with a racket)	Mini‐hurdle sprint (jump over small hurdles like a speedy rabbit)
Activity 3	Trampoline play (jumping while mimic or hold favorite stuffed toy)	Vaulting box challenge (jump over the vaulting box like a superhero)	Ball games (aim and throw at target numbers)
Cool‐down	Stretching exercises	Same as Example 1	Same as Example 1
Equipment cleanup	Children help tidy up	Same as Example 1	Same as Example 1
